# Community-based mental health services in Brazil

**DOI:** 10.17650/2712-7672-2020-1-1-60-70

**Published:** 2020-09-02

**Authors:** Denise Razzouk, Daniela Cheli Caparroce, Aglae Sousa

**Affiliations:** Centre of Mental Health Economics, Department of Psychiatry, Universidade Federal de Sao Paulo (Unifesp)

**Keywords:** community mental health services, Brazil, health policy, mental disorders, mental health, developing countries, health resources, амбулаторная психиатрическая служба, Бразилия, политика в сфере здравоохранения, психические расстройства, развивающиеся страны, медицинские ресурсы

## Abstract

**Introduction.:**

The shift from the hospital-based model of care to community-based mental health services began three decades ago and is still an ongoing process in Brazil.

**Objectives.:**

To update data on the development of the community mental health services network in Brazil in relation to service availability and structure, manpower, pattern of service use, financing, epidemiological studies and the burden of mental disorders, research and national mental health policy.

**Methods.:**

Searches were constructed to collect data on indexed databases (Medline, Scielo), as well as governmental,NGOs and medical council sources, reports and the grey literature up until 30th March, 2019.

**Results.:**

Community mental health services are unevenly distributed in the country. Brazil leads the world in terms of the prevalence of anxiety disorders, ranking fifth for depression prevalence. Violence and suicide rates are two growing factors which exacerbate the prevalence of mental disorders prevalence. An increased reduction of the number of psychiatric beds in the country, in addition to the unbalanced growth of services in the community, has resulted in treatment gaps and the underutilization of services and barriers to treating people with the most severe psychosis. Investment in mental healthcare is still scarce. However, mental health funding is not addressed according to the population´s needs and scientific evidence, resulting in a waste of resources and inefficiency. Programmes and service interruptions are common according to each government mandate.

**Conclusion.:**

Successive changes in ideological perspectives have led to the introduction of policies which have caused fragmentation in the mental health system and services. A lack of evaluation and transparency of services and costs are the main barriers to integrating multiple services and planning long-term developmental phases.

## INTRODUCTION

Brazil is an upper-middle-income country according to the World Bank classification, located in South America, with a population of nearly 215 million inhabitants and a life expectancy of 76 years. Income per capita is $8,600 with a GINI coefficient of 0.51. The Federal Brazilian Constitution of 1988 adopted the Social Welfare State, allowing for social rights including free access to education and health.

The Brazilian Universal Health Coverage (UHC) was created in 1988, targeting three key Unified Health System's (SUS) principles: universal free access to health services, equity and comprehensive healthcare. The Federal Brazilian Constitution pledges health as a universal right that should be guaranteed by the state. Before the UHC's implementation, only employed people received healthcare, that is, 21% of the population. In 1990, the law 8080 was enacted, assuming a broader concept of health rather than the absence of disease, but also aiming to promote wellbeing, quality of life and inequity reduction.

Multiple reports on the abuse and mistreatment of patients in psychiatric hospitals during the 70s and '80s have encouraged the closure of such institutions. Although mental healthcare was included in the SUS in 1988, treatment was mainly delivered under a contract between the government and private psychiatric hospitals, to which 93% of the mental health budget was directed [1].

The Brazilian Mental Health Policy was established primarily after the Declaration of Caracas in 1990 [2], based on patients' human rights and delivering mental healthcare in the community [1]. However, the effective reduction in the availability of psychiatric beds occurred after Mental Health Law Number 10.216 was enacted in 2001 to replace hospital-centred care with community mental healthcare [3].

The objective of this manuscript is to update data on the progress of developing community mental health services in Brazil and to discuss the advances, challenges and limitations related to the implementation of national mental health policies.

## MATERIAL AND METHODS

We constructed the searches to find information on the following topics: a) brief historic aspects of the establishment of community mental healthcare in the country, b) description of the main characteristics of community mental health services, c) general characteristics of the mental health system in terms of manpower, costs, financing, beds, treatment and service availability, d) status of mental disorders in the country in terms of prevalence, burden and accessibility to services, e) strengths and weaknesses of the mental health system and policies. Searches were developed to retrieve relevant studies, documents, reports and government databases on national and international databases such as Medline, Scielo, Google Scholar, the Ministry of Health of Brazil website and World Health Organization website up until 30th March, 2019. Articles were selected and included in this overview, according to the relevance and completeness of the information regarding the descriptions and data related to the aforementioned topics. It was not an exhaustive search and it may be missing some related publications.

## RESULTS

### 1. Description of the provision of community mental health services

The shifting from the hospital model to a community mental health model has been developing over the past few decades, characterized by a dramatic reduction in the number of psychiatric hospitals from 87,134 beds in 1994 to 25,097 in 2016 [3]. The "Programa Nacional de Avaliagao dos Servigos Hospitalares-PNASH"- a programme to evaluate psychiatric hospitals - was created to assess all psychiatric beds available in the public health system and to cancel any public-private partnership should psychiatric hospitals not meet certain requirements, such as hospital size and number of beds, inappropriate structure and conditions, reports of abuse or human rights infringements and electroconvulsive therapy use, even for eligible patients. This programme was addressed to assess the user' s satisfaction with the services, to establish indicators for the health service's performance, to implement quality standards of care and to support health managers.

Although Law Number 1631/2015 established the ideal rate of 0.45 psychiatric beds per 1,000 inhabitants [4], the coverage for psychiatric hospitalization dropped by 40% - from 0.22 to 0.12 per 1,000 inhabitants - between 2005 and 2016, leading to a shortage of psychiatric beds in the country. During this period, there was a tiny increase in the number of psychiatric beds in general hospitals, from 570 in 2013 to 1,117 beds in 2017 [5]. Moreover, there was an uneven distribution of psychiatric beds across the country: 0.012 per 1,000 inhabitants in the northern region and 0.18 per 1,000 inhabitants in the southern region [6].

The Brazilian community mental health system comprises a complex psychosocial network of mental health services ("Rede de atengao psychosocial -RAPS"), created in 2011, with the aim of preventing, treating and promoting the social inclusion of people suffering from mental illness and drug misuse. Recently, a new ministerial ordinance included other services in the RAPS, such as mental health outpatient services (ambulatory), drug rehabilitation centres ("Comunidades terapeuticas") and psychiatric and day hospitals [7]. In Table 1, all available mental health services are described [8]. People with mild to moderate mental health problems are treated in primary care by the general practitioners under the supervision of mental health specialists. People with moderate to severe mental disorders are treated by mental health specialists in outpatient services - specialized polyclinics with other medical specialities. People with psychosis, alcohol and drug disorders, autism and other severe mental disorders are treated in different types of Centres of Psychosocial Care - CAPS described in Table 1 [6]. Therefore, mental health services are integrated into the entire health system. Referrals to mental health services come from the primary care units, first-aid care, emergency care, hospitals, as well as self-referrals.

The growing number of community mental health services increased from 424 Centres of Psychosocial Care (CAPS) in 2004 to 3,013 in 2018. The number of CAPS per 100,000 inhabitants increased from 0.21 in 2004 to 0.86 in 2014 [6]. According to the Ministry of Health's indicators, a rate of between 0.5 and 0.69 CAPS per 100,000 inhabitants is considered good coverage and rates in excess of this are regarded as excellent coverage. According to the last report in 2015, CAPS are unequally distributed across the country, ranging from 0.38 to 1.57 CAPS per 100,000 inhabitants [6].

**Figure 1:**
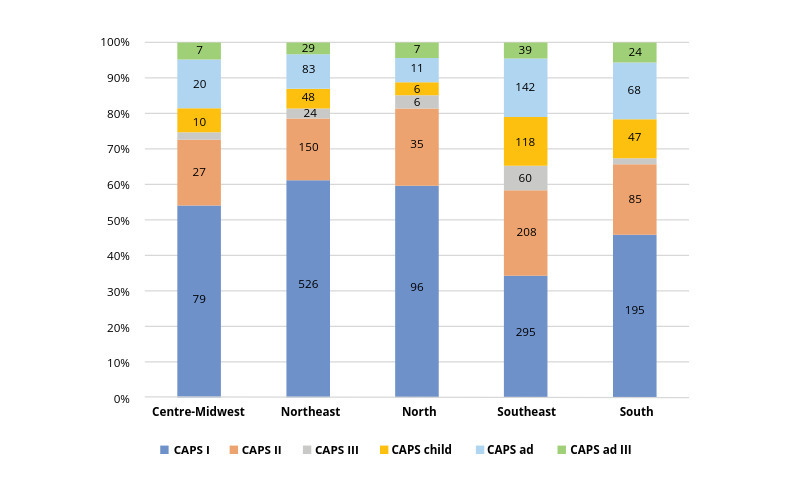
Figure 1. Distribution of Centres of Psychosocial Care in Brazil

**Figure 2:**
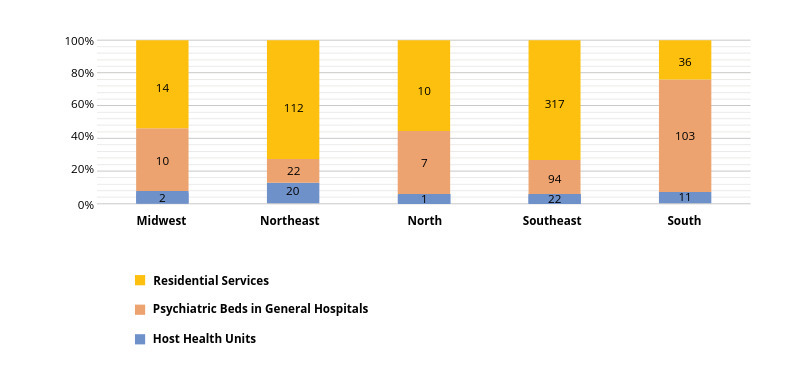
Figure 2. Distribution of host health units, residential services and general hospitals with psychiatric beds in Brazil, 2017

There is an unequal distribution of community mental health services across the country as shown in Figures 1‒2. The majority of CAPS services are concentrated in the South and in the southern half of the country, where 56% of the population lives. The majority of mental health services are located in the state of Sao Paulo, in the southeast of Brazil. This area accounts for 21% of the Brazilian population and 34% of Brazil' s GDP. Psychiatric beds in general hospitals are still scarce in the country and they are mostly concentrated in the South and southern regions (Figure 2). Residential facilities have also been created to accommodate patients discharged from psychiatric hospitals. Each residential service is located close to one CAPS, where mental health treatment is provided. In 2005, 9,000 people were living in Brazilian psychiatric hospitals and in 2014, 4,439 people were still living in 53 psychiatric hospitals in the state of Sao Paulo, in which long-term hospitalization was concentrated [9].

**Table 1:** Table 1. Description of services provided by the Brazilian Mental Health Network

Сharacteristics	Description	Number of services
CAPS-1	Centre of Psychosocial Care for moderate and severe mental disorders and drugs misuse in a city with over 15,000 inhabitants.	1069
CAPS II	Centre of Psychosocial Care for moderate and severe mental disorders and drugs misuse in a city with over 70,000 inhabitants.	476
CAPS III	Centre of Psychosocial Care for moderate and severe mental disorders and drugs misuse in a city with over 150,000 inhabitants. Includes five psychiatric beds.	85
CAPS ADII	Centre of Psychosocial Care for moderate and severe drugs misuse in a city with over 150,000 inhabitants.	69
CAPS AD III	Centre of Psychosocial Care specifically focused on alcohol and drug misuse, with eight to 12 night beds.	309
CAPS AD IV	Centre of Psychosocial Care for severe alcohol and drug misuse, providing 24-hour mental healthcare in a city with a population greater than 500,000 inhabitants. The number of psychiatric beds ranges from eight to 30.	-
CAPS child	Specifically for children and adolescents with severe mental illness (such as autism).	201
Host health units for adults suffering from drug misuse	Accommodation in houses, with support from professionals for extremely vulnerable crack and other drug users with limited family and social support. Short stays up to six months. (known as “Unidades de Acolhimento”)	34
Host health units for children	Accommodation in houses with support from professionals for extremely vulnerable children and adolescents between the ages of 12 and 18 years, suffering from alcohol or drug misuse and with limited social support. (known as “Unidades de Acolhimento”)	22
Day hospital care	Specific mental health treatment for certain patients recently discharged from hospital and with intensive care needs (up to 12 hours per day).	649
Psychiatric unit in general hospital	Brief hospitalization for people with acute symptoms, risk of suicide, severe self-care impairment, aggressive behaviours. There are 233 general hospitals with 1,167 psychiatric beds in the country.	233
Drug rehabilitation centres	(“Comunidadesterapêuticas”) Mid-term stay in houses under the supervision of professionals focused on helping patients with alcohol and drug dependence to recover and support them with rehabilitation.	412
Residential service	Housing for patients discharged from long-stays in psychiatric hospitals with minimal or no family support. Two mental health carers provide supervision for up to eight residents.	3470
Psychiatric hospital	Psychiatric, specialized hospitals delivering care outside general hospitals (approximately 25,000 beds)	167
Outpatient care	Specialized outpatient care including psychiatry and other medical specialities for treating moderate and severe mental disorders and other comorbidities.	1991

### 2. Service use, mental health needs and population characteristics

#### 2.1. Prevalence and burden of mental disorders in Brazil

Mental disorders account for 13% of DALYs in Brazil, and depression is the fourth leading cause of this burden. In the Americas, Brazil leads the years lived with disability (YLD), with mental disorders accounting for 36% [10]. Brazil has the most cases of anxiety disorders globally and ranks fifth in terms of the number of cases of depression [11]. Anxiety and depression were the fifth and sixth leading causes of YLD in Brazil's GBD 2016 study [11], accounting for 7.5% and 9.3% of YLD [10]. Schizophrenia and bipolar disorders accounted for 1.6% and 1.4% of YLD in Brazil [10].

On the other hand, alcohol and interpersonal violence was the second leading cause of YLD in Brazil in 2016 [12]. Moreover, the behavioural risk factor for alcohol and drug use has almost doubled between 1900 and 2016, accounting for 12.2% of DALYs, and is the leading risk factor among males [12]. In relation to violence and traumatic events, one study [13] in Rio de Janeiro and Sao Paulo has shown a prevalence of traumatic events of 35% and 21%, respectively, and these events were associated with psychiatric disorders, indicating the relevance of social, environmental and cultural factors affecting mental health.

Moreover, suicide is the fourth leading cause of mortality among people between the ages of 15 and 29 [14,15]. The Brazilian suicide rate was 5.8 deaths per 100,000 inhabitants in 2014 [14]. Women account for 69% of suicide attempts, 58% of them by poisoning. More than 60% of suicide occurred in the South and southern regions of the country, which are the wealthiest regions. The suicide rate is much greater among men than women, especially among older men but also among adolescents: 9.0 per 100,000 males and 2.4 per 100,000 females. The suicide rate among adolescents has increased by 24% within a decade and has been associated with social inequality and unemployment [16,17]. It is also the leading cause of mortality among the indigenous population between the ages of 10 and 19.

Despite the high prevalence of mental disorders in Brazil, mental health services are underused. Some obstacles hinder the effective implementation of community mental health services. For instance, there is an important communication gap between mental health policies and the population, in relation to the awareness of mental health symptoms and the availability of treatment. One study [18] has shown that these services were used by 10% of people suffering from anxiety disorders, 22% of people with depression and 34% with both disorders. Service use was associated with white people older than 30, with low resilience and living in low homicide rate regions. Another study [9,19] has shown that only 15% of people with psychotic disorders, discharged from psychiatric hospitals and living in residential services, received psychosocial interventions from community mental health services, regardless of their need for psychosocial rehabilitation.

#### 2.2. Length of time in psychiatric hospitals

The reduction of psychiatric beds and the acceleration of the deinstitutionalization process has resulted in a shortage of psychiatric beds, as well as a reduction in the length of time spent in psychiatric hospitals. The annual admissions per 100,000 inhabitants for a psychiatric hospital and general psychiatric bed unit were on average 216 and 60.8, respectively [20]. The length of time of hospitalizations was less than one year in 63% of admissions, and financial incentives were offered to shorten the length of time of hospitalizations [6].

#### 2.3. Quality of mental healthcare

Mental health policies tend to focus on reducing the length of hospitalization and monitoring the coverage and access to services, as well as the number of patients seen by doctors, rather than evaluating treatment goals and the effectiveness of treatments. There are some isolated initiatives to improve the technical efficiency of services, however, mental health indicators are not used in the country. Health cost management is still challenging and an evaluation of wasted resources is not routinely taken into account.

2.3.1. Staff

There is a shortage of mental health professionals in Brazil, especially in the poorest regions of the country. In 2014, the ratios of mental health professionals per 100,000 inhabitants were 3.40 psychiatrists, 3.22 psychologists, 13.99 social workers, 3.05 nurses, 1.16 occupational therapists and 4.15 were made up of other doctors [20]. According to the last Brazilian Medical Council survey [21], there were 10,396 psychiatrists in the country, corresponding to 5.01 per 100,000 inhabitants, a 30% increase over the last five year period, although the need for psychiatric staff is estimated to be double this number. However, psychiatrists account for 2.7% of all medical specialities in the country but the distribution of professionals is still unequal across different regions: 2.1% in the North, 7.8% in the Midwest, 12.6% in the Northeast, 23.1 % in the South and 53.4% in southern regions. Therefore, the variation of the number of psychiatrists in the country ranges from 0.69 to 12.84 per 100,000 inhabitants.

2.3.2. Medication and mental health interventions

Psychotropics are subsidized primarily by the federal government, especially those drugs with a high cost. In this regard, atypical antipsychotics correspond to 93% of public expenses in terms of high-cost drugs. There are 14 antipsychotics available in the SUS, five of which are atypical antipsychotics. There is a shortage of studies assessing the cost-effectiveness and value of these drugs in the country and a huge variation in prices, even from the perspective of the government [22]. On the other hand, fewer antidepressants are available in the health system, despite their lower price and a high prevalence of depression and anxiety in the country [23]. Lithium carbonate, anticonvulsants and anticholinesterases and benzodiazepines are also available in the public health system. Only one guideline has been implemented to regulate the use of antipsychotics in treating schizophrenia and bipolar disorders.

One study [24] has found a 6.5% incidence of psychotropic use among Brazil's general population, with a female/male ratio of 3:1: antidepressants (2.7%), anorectics (1.65%), tranquillizers (1.61%) and mood stabilizers (1.23%). General practitioners issued 46% of such prescriptions and 86% of these drugs were paid for by the patient and family.

Non-medical mental health interventions vary in terms of type, quantity and quality and they are delivered at all levels of care. One study [25] evaluated 10 CAPS in the city of Sao Paulo and identified 457 different mental health activities, highlighting a wide range of heterogeneity among these services. Educational programmes, such as 'Unplugged' or the Brazilian version Tamojunto' were implemented to prevent the use of alcohol and drugs among adolescents, although, the outcome was the converse of expectations, resulting in an increase of 30% in terms of the risk of consumption of such drugs in this population [26]. Recently, the programme was cancelled and, for the first time, the current Ministry of Health is financing an effectiveness study using scientific evidence to further analyse the appropriateness of this programme in the country.

2.3.3. Social inclusion

Some programmes aim to promote the social inclusion of people with mental health disorders in the community. In this regard, the "Return Home Programme" ("De voltapara casa"), created in 2003 by the Ministry of Health, consists of a monthly financial benefit (BRL 412.00), offered to 4,349 people discharged from long-term hospitalization in psychiatric institutions and returning to live with their families [6]. The annual costs of this programme are, on average, BRL 20 million.

In 1991, a law advocating inclusion in the workplace of people with any type of disability was enacted, although it was not specified which type of intellectual deficiency was included. In this regard, an enterprise with 100 employees or more should employ between 2 and 5% of people with a physical or intellectual deficiency. The term mental deficiency was included in Law 8213/91, in 1999, however, the majority of people with mental disorders remained unemployed. In 2012, the complementary Law 12.764, extended this right to people on the autism spectrum, however those with schizophrenia were not covered by these laws.

2.3.4. Financing

Health expenditure corresponded to almost 9% of Brazilian GDP in 2015, of this 5.2% was spent by families and private institutions [27]. One-third of the population has private health insurance and spends on average BRL 440.00 per month.

Public health expenditure in Brazil corresponded to 3.8% of GDP in 2018, and the federal government invested 1.8% of GDP in health in 2017. The federal government spent BRL 117 billion on health, of which two- thirds were intended for medium and high complexity level care (secondary and tertiary) and the remaining third for primary care and medication [28]. On average, annual public health expenditure per capita is estimated at around BRL 1,200.00. Health expenditure is also funded by the states and municipalities, respectively, corresponding to 12% and 15% of their revenues.

There are no official data available concerning the mental health budget in Brazil, although previous studies have estimated the percentage of the health budget allocated to mental health, to be around 2 to 2.5%; the Atlas survey of 2014 reported a federal investment of $ 43.16 per capita in mental health [20]. In 2017, the Brazilian Ministry of Health assigned daily tariffs to psychiatric hospitals (up to 90 days) according to the number of psychiatric beds: BRL 82.40 per bed per day (up to 160 beds), BRL 70.00 (between 160 and 240 beds), BRL 63.11 (between 241 and 400 beds) and BRL 59.00 (more than 400 beds) [29]. It also created a new service, CAPS AD IV; the monthly investments for implementing these services varied from BRL 33,000.00 to BRL 99,000.00 according to the number of psychiatric beds.

Few studies are aimed at estimating the direct costs of mental health services and treatments. One study estimated the costs of 20 residential services in Sao Paulo and found the mean annual costs per resident to be $19,621.44 in 2017 [9,30], corresponding to double the Brazilian per capita income. The federal government financed 73.4% of these costs and the remainder was funded by local government. Residential services corresponded to 90% of the total package of care for patients with mental disorders, discharged from long stays in psychiatric hospitals. Residential services costs were related to geographical regions and to the length of time residents lived in psychiatric hospitals and used residential services [9]. The mean and standard deviation of psychotropic costs per month and per resident in this sample was BRL 216.07±380.40 for the year 2011. Psychotropic costs were mainly influenced by atypical antipsychotics polytherapy use, and the latter was the main cost driver of direct health costs [19].

Another study relating to the estimation of CAPS-AD costs in a city located in the state of Sao Paulo, found that of the monthly cost of BRL 64,017.54, 62.1 % was funded by the federal government [31,32]. On average, direct health costs per capita of patients with alcohol and drug dependence in this service were $149.00 per month.

On the other hand, the federal government funded 20% of direct health costs (medication and clinical treatment) of daily psychiatric hospitalization, excluding hotel costs [33]. On average, the daily psychiatric hospitalization costs were BRL 371.00 per capita for the year 2015, with hotel costs accounting for half of these costs.

## DISCUSSION

Few studies have evaluated mental health services in Brazil; one systematic review identified 35 studies, all focused on the South and southern regions [34]. The main findings of this review showed some advances in relation to patients' participation in CAPS' activities, good crisis management and a high level of family satisfaction with services. In contrast, mental health professionals exhibited dissatisfaction with regard to intense workload and high demand, a lack of professional skills and training, and unsatisfactory work conditions.

Moreover, the lack of integration of mental health services and primary care was raised in this review and other publications [34,35], emphasizing the need to overcome barriers, such as a lack of professional training, a lack of professionals within primary care, obstacles to referral and counter-referral among services and no clear policies directed towards the health and related sectors.

Psychiatric reform in Brazil was mainly influenced by Basaglia' s ideological views, allowing some policymakers to establish mental health policies, refusing the need for hospitalizations in cases of acute episodes with moderate to severe risks. Therefore, policies were not based on scientific evidence and population needs. In this regard, the accelerated reduction of psychiatric beds without appropriate community support caused harm to those who remained without access to treatment. The number of psychiatric hospitals and psychiatric beds in general hospitals is still insufficient to cover demand [36]. Instead, the number of psychiatric beds available in the country accounts for half of those in high-income countries. The Ministry of Health has been alerted to the fact that 50,000 people with severe mental disorders are in prison as a consequence of this rapid psychiatric reduction and inefficient strategies for treating people discharged from hospitals [7,37]. There is also an important treatment gap in cases of anxiety and depression disorders in the country, although these are highly prevalent in primary care. Estimates of 12-month anxiety prevalence have shown that 23% of people with anxiety in the city of Sao Paulo received treatment, and 10% of them received adequate treatment [38]. Despite the high prevalence of symptoms of these disorders in primary care ranging between 51% and 64% [39], no specific programme or policy is targeting them. The main obstacles in many regions of the country are lack of service access and trained professionals to provide appropriate treatment [40]. Treatment for these disorders is efficacious, cost- effective [41] and financially feasible [23], although mental disorders have not been a priority, despite the high numbers of people suffering from such disorders.

Mental healthcare research is still scarce in terms of the lack of information relating to services evaluation, treatment effectiveness and health costs [34]. There is no tradition in the country for decision making to be based on national scientific evidence when drawing up health policies. CONITEC (Comissao Nacional de Incorporagao de Tecnologias), a committee for health technologies' assessment, has established that cost- effectiveness studies are one of the requirements when considering the inclusion of new treatment in the public system. However, these studies are modelling studies, based on international data and conducted by pharmaceutical companies with clear conflicts of interest. There are few empirical data on the costs and effectiveness of treatments and services, and the majority of policymakers and decision makers are not trained in health economics. A study among stakeholders identified 10 priorities in mental health research, such as studies on the cost-effectiveness of antipsychotics, evaluation of specific interventions for alcohol and drug use and qualitative studies exploring the barriers to mental health treatment and services [42].

## FUTURE DEVELOPMENTS AND CONCLUSION

The implementation of community mental healthcare is still an ongoing process, with adjustments being made according to a heterogeneous perspective, with every new government mandate. The lack of a long-term plan of action for mental healthcare leads to service and programme interruptions and consequently, results in a waste of resources each time a new mandate starts.

A lack of transparency on services information and costs is the main barrier to further developments. There is a need for integrating multiple services and for planning long-term developmental phases [15]. Moreover, combining the evidence resulting from national research and mental health policies should be aligned to address the needs of vulnerable groups.

## Acknowledgements

Thanks to Jair de Jesus Mari for his comments and contributions to this manuscript.

## Author Contributions

Denise Razzouk participated in the planning, searching, retrieval and selection of data, as well as the writing of the paper. Daniela C. Caparroce participated in the searching and retrieval of data, as well as the writing of the paper. Aglae Sousa participated in the searching and retrieval of data, as well as the writing of the paper.

## Funding

The authors declare that there was no funding for this work.

## Conflict of Interest

The authors declare no conflict of interest.

## References

[webpage-ref-f5d00b7ed50d9949dfb72ca19192a653] (2005). Reforma psiquiatrica e politica e saúde mental no Brasil [Psychiatric reform and the mental health policy in Brazil]. Internet.

[conference-paper-ref-cad24132d76e85aad9d33bf9359d6636] Bolis M, PAHO/WHO (2002). The Impact of the Caracas Declaration on the Modernization of Mental Health Legislation in Latin America and the English-speaking Caribbean. http://citeseerx.ist.psu.edu/viewdoc/download?doi=10.1.1.525.8287&rep=rep1&type=pdf.

[journal-article-ref-34bf6bf41fe5d2cef8d45f5d7028a9e1] Kilsztajn S, Lopes E, LimaI L (2008). Hospital beds and mental health reform in Brazil. Cad Saúde Pública.

[webpage-ref-dacd9923f5346297e11925faeb9faed1] (2017). Em 11 anos, o SUS perde quase 40% de seus leitos de internação em psiquiatria.. [ In 11 years, SUS lost 40% of psychiatric beds] [In Portuguese]. Brazil, Federal Medical Council (Conselho Federal de Medicina do Brasil).

[webpage-ref-8635a6ca927387412717c49fc5eb9e7a] (2017). Panorama e Diagnóstico da Política Nacional de Saúde Mental. [Overview and diagnostic of National Mental Health Policy] [In Portuguese]. Ministério da Saúde do Brasil. [National Mental Health Policy Review. Ministry of Health of Brazil].

[webpage-ref-113b08237462949f33a75de2bac012ff] (2015). Saúde Mental em dados 12. Ministério de Saúde do Brasil. [Mental Health in Data. Ministry of Health of Brazil].

[webpage-ref-3e191e58d2c18a76a8221fcd2f100ba9] (2019). Nota Técnica 11/2019-CGMAD/DAPES/SAS/MS. [Technical Note 11/2019-CGMAD/DAPES/SAS/MS]. Brazil, Ministério da Saúde [Ministry of Health Brazil].

[journal-article-ref-00ea9779bff7235c38162aef47c3cd03] Mateus M D, Mari J J, Delgado P G (2008). The mental health system in Brazil: Policies and future challenges. Int J Ment Health Syst.

[webpage-ref-2fcaa14f0b8551e961c9f3f6cad2acb8] PAHO (2018). The Burden of Mental Disorders in the Region of the Americas, 2018.. Internet.

[journal-article-ref-eb20c02aab2585282df7e55e71bdca46] Sousa I (2017). Brazil: the world leader in anxiety and depression rates. Brazilian Psychiatric Journal.

[journal-article-ref-46b14f6067fed1a5beb2d3845eb87522] Marinho F (2018). Burden of disease in Brazil, 1990–2016: a systematic subnational analysis for the Global Burden of Disease Study 2016. The Lancet.

[journal-article-ref-e34fa1a56d8211c4dbb6e09b3eb51bf8] Ribeiro W, Mari J, Quintana M (2013). The Impact of Epidemic Violence on the Prevalence of Psychiatric Disorders in Sao Paulo and Rio de Janeiro, Brazil. PLoS ONE.

[webpage-ref-31f0aff3529d78dedde6b89aab4b415b] (2017). Brazil, Ministério da Saúde, Secretaria da Vigilancia em Saude. [Ministry of Health of Brazil, Secretariat of Health Surveillance]. Perfil epidemiológico das tentativas e óbitos por suicídio no Brasil e a rede de atenção à saúde [Epidemiological profile of suicide attempts and deaths in Brazil and a health care network]. Boletim Epidemiológico [Epidemiological Bulletin].

[journal-article-ref-30234281c58216d1d2151fb23e9b6260] Thornicroft G, Alem A, Antunes Dos S R (2010). WPA guidance on steps, obstacles and mistakes to avoid in the implementation of community mental health care. World Psychiatry.

[journal-article-ref-7a3d752826638349c3e9ce7b7d9fa6b3] Jaen-Varas D, Mari J, Asevedo E (2019). The association between adolescent suicide rates and socioeconomic indicators in Brazil: a 10-year retrospective ecological study. Brazilian Journal of Psychiatry.

[journal-article-ref-f77c4e0a2c818bafb83f97f4b7e70c07] Asevedo E, Ziebold C, Diniz E, Gadelha A, Mari J (2018). Ten-year evolution of suicide rates and economic indicators in large Brazilian urban centers. Curr Opin Psychiatry.

[journal-article-ref-80f740e982ec8921c397abfac30501e9] Filembaum G, Blay S, Melo (2019). Use of mental health services by community-resident adults with DSM-IV anxiety and mood disorders in a violence-prone area: São Paulo, Brazil. Journal of Affective Disorders.

[journal-article-ref-7936e3ed92f988b67557228a95e89e57] Razzouk D, Kayo M, Sousa A (2015). The impact of antipsychotic polytherapy costs in the public health care in Sao Paulo, Brazil. PlosOne.

[journal-article-ref-14fb3241c01441762cdc2e18e95e9cc6] Razzouk D (2019). Accommodation and Health Costs of Deinstitutionalized People with Mental Illness Living in Residential Services in Brazil. Pharmacoeconomics Open.

[webpage-ref-1773f6c40bd7abee8b3b7d4c5b3a8c70] (2020). WHO. Mental Health Atlas 2014: Brazil profile. World Health Organization. 2014. https://www.who.int/mental_health/evidence/atlas/profiles-2014/bra.pdf?ua=1.

[book-ref-f9a2512b289782bd6ce711ed14f54215] Scheffer M (2018). Demografia Médica no Brasil 2018. [Medical demographic in Brazil].

[journal-article-ref-2a168ef11843698a0802c707662af5db] Razzouk D (2017). Cost variation of antipsychotics in the public health system in Brazil: the implication for health resource use. J Bras Econ Saúde.

[journal-article-ref-cc1b9a298d9978431a42791b50fd12e4] Razzouk D (2016). Why should Brazil give priority to depression treatment in health resource allocation?. Epidemiol Serv Saude.

[journal-article-ref-0b221f28b0e95b07d86eb31378c0105e] Quintana M, Andreoli S, Moreira F (2013). Epidemiology of Psychotropic Drug Use in Rio de Janeiro, Brazil: Gaps in Mental Illness Treatments. PLoS ONE.

[journal-article-ref-a16caef507ddf1dd63c7b55c0083a122] Nascimento A, Galvanese A (2009). Evaluation of psychosocial healthcare services in the city of São Paulo, Southeastern Brazil. Rev Saúde Pública.

[journal-article-ref-d340967890ffadbd4bff0676431ce881] Sanches Z, Sanudo A, Andreoni S, Schneider D, Pereira A, Faggiano F (2016). Efficacy evaluation of the school program Unplugged for drug use prevention among Brazilian adolescents. BMC Public Health.

[webpage-ref-694d6d024383b9e6943209cf06dbc441] (2018). National Health Council, CNS. Gastos dos brasileiros com saúde [Brazilian health expenditures ].

[webpage-ref-a94eb4ec34ae34bc0460b75f7a30f12a] (2018). Aspectos fiscais da saúde do Brasil [Fiscal aspects of health in Brazil]. Brazil, Ministerio da Fazenda. [Ministry of Finance of Brazil].

[webpage-ref-2030b86c546191c19257b033e56e54c5] (2017). Law 3.588, 21st December 2017. Ministry of Health Brazil (MS-Brazil).

[book-ref-b3eacbb1b4250c082dd601f0c27a93a2] Razzouk D, Razzouk D (2017). Estimating costs of residential services. Mental Health Economics: The costs and benefits of psychiatric care.

[book-ref-573da6facf87a48cdf21f769bc26d61f] Becker P, Razzouk D, Razzouk D (2017). Estimation of cost for community mental health services.

[journal-article-ref-0c649c70cf5f3f56a8c1a60e5904449b] Becker P, Razzouk D (2018). Cost of a community mental health service: a retrospective study on a psychosocial care center for alcohol and drug users in São Paulo. Sao Paulo Medical Journal.

[book-ref-b1f778872693f4ca4b0465222c88eb9e] Siomi A, Razzouk D (2017). Costing psychiatric hospital wards in general hospitals. In: Razzouk D, editor. Mental Health Economics: The costs and benefits of psychiatric care.

[journal-article-ref-1affbcc3376b9dc2fb15c6ff4b0db5d5] Costa P, Colugnati F, Ronzan T (2015). Mental health services assessment in Brazil: systematic literature review. Ciênc Saúde Coletiva.

[journal-article-ref-ebaff451c76d43a0c02d7b8a0e51c7a6] Mari J J (2014). Mental healthcare in Brazil: modest advances and major challenges. Advances in psychiatric treatment.

[journal-article-ref-dad6f3a54369de40c26ba76ef14ff294] Loch A, Gattaz W, Rossler W Mental healthcare in South America with a focus on Brazil: past, present, and future. Curr Opin Psychiatry.

[journal-article-ref-88a187f573694123dbda17ddbfdec5b4] Andreoli S, Santos M, Quintana M (2014). Prevalence of Mental Disorders among Prisoners in the State of Sao Paulo, Brazil. PlosOne.

[journal-article-ref-f1e780eb96762b54303f36b0e763bf87] Alonso J, Liu Z, Evans-Lacko S (2018). Treatment gap for anxiety disorders is global: Results of the World Mental Health Surveys in 21 countries. Depress Anxiety.

[journal-article-ref-1b220b8f16d8c3070eca14818ee8eacf] Gonçalves D, Mari J J, Bower P (2014). Brazilian multicentre study of common mental disorders in primary care: rates and related social and demographic factors. Cad Saúde Pública.

[journal-article-ref-a531faf9ea245efb9a93db402fd41649] Heidt R (2016). Prevent Depression: Improving Access to Brazil's Mental Health Services. Clinical Social Work and Health Intervention.

[journal-article-ref-7f7e3b19a0f972b46cf84ffa89f92c44] Chisholm D, Sweeny K, Sheehan P et al. Scaling-up treatment of depression and anxiety: a global return on investment analysis. Lancet Psychiatry 2016;3(5).

[journal-article-ref-f123c50482a2ae6f11eaa1fb7a2fe1ef] Gregorio G, Tomlinson M, Gerolim J (2012). Setting Priorities for Mental Health Research in Brazil. Brazilian Psychiatric Journal.

